# COVID-19 pandemic and the widening gap to access cancer services in Uganda

**DOI:** 10.11604/pamj.supp.2020.35.140.25029

**Published:** 2020-08-10

**Authors:** Derrick Bary Abila, Provia Ainembabazi, Henry Wabinga

**Affiliations:** 1 Makerere University College of Health Sciences, Kampala, Uganda,; 2 Faculty of Biology, Medicine and Health, University of Manchester, Manchester, UK,; 3 Infectious Diseases Institute, Makerere University College of Health Sciences, Kampala, Uganda,; 4 Kampala Cancer Registry, Department of Pathology, Makerere University College of Health Sciences, Kampala, Uganda

**Keywords:** Cancer services, COVID-19, cancer control, Uganda

## Abstract

The COVID-19 pandemic and its public health control measures have led to worldwide interruptions in healthcare service delivery, and cancer services are no exception. These interruptions have exacerbated the effects of previously reported barriers to accessing cancer care which was reportedly low even before the pandemic. If these effects are not mitigated, the achievements in cancer control that had already been made could be watered down. Measuring the impact of COVID-19 pandemic control measures on delivery of and access to cancer services in Uganda as well as other countries worldwide can inform the design of current and future responses to epidemics while putting into context other diseases like cancer that have a high burden.

## Commentary

Worldwide, Coronavirus Disease 2019 (COVID-19) (SARS-CoV-2) cases have increased rapidly since the first case was reported in December 2019 in Wuhan City, Hubei Province, China [1]. As of 16 July 2020, there were 13,378,853 cases, reported from all continents along with 580,045 deaths [2]. The World Health Organization (WHO) declared the situation to be a pandemic on 11 March 2020. Uganda reported its first case of COVID-19 on 21 March 2019, and as of 16 July 2020, 1,043 cases of COVID-19, with 1,014 recoveries and zero COVID-19-associated deaths have been reported [2]. The COVID-19 pandemic has led to worldwide interruptions in healthcare service delivery, and cancer services are no exception. The Uganda Cancer Institute (UCI) is an autonomous body, with the mandate by the Uganda Cancer Institute Act of 2016 to, among others, undertake and coordinate the prevention and treatment of cancer and cancer-related diseases in Uganda. UCI also serves as a Center of Excellence for Oncology in East Africa, and it is the only facility that can provide comprehensive cancer services in Uganda [3]. Estimates of the burden of cancer in Uganda and the impact of cancer control strategies have previously been measured by cancer registries in Uganda like the Kampala Cancer Registry. This is a population-based cancer registry that serves the population of Kyadondo County comprised of Wakiso district and Kampala, the capital of Uganda.

The cancer burden is rising in Uganda; according to the 2018 GLOBOCAN estimates, over 32,617 new cases and 21,829 death related to cancer were estimated to have been registered in Uganda in 2018. The most common cancers among women are cervical cancer and breast cancer, with incidences of 50.2 and 31.2 per 100,000 of the population, respectively. Prostate cancer is the most common cancer among men, with an incidence of 58 per 100,000 of the population. Kaposi Sarcoma, lung cancer, and liver cancer also contribute to the burden of cancer in this population [4]. Among children under 18, the most common cancers reported are Burkitt´s lymphoma, Kaposi Sarcoma, non-Burkitt´s lymphoma non-Hodgkin lymphoma, acute lymphoblastic leukemia, and Wilms tumor [5]. The individual and healthcare burden posed by these cancers can be reduced through cancer preventive services, including early diagnosis through routine screening, adherence to treatment, and public health campaigns like those that promote engaging in physical activity and eating a healthy diet. These essential functions to reduce the burden of cancer in Uganda, and the East Africa region, are being affected by the current COVID-19 lockdown measures, restrictions on travel, and suspension of outreach and screening services [6,7].

The COVID-19 lockdown measures implemented in Uganda are based on WHO-issued guidance and include the use of restrictive physical distancing to limit the spread of the virus. In this paper, we focus on restrictions of public and private transport, restriction on income-generating activities, observance of person-to-person physical distancing of 2-4 meters, and observance of a daily curfew because they could influence the delivery of and access to cancer care services [7]. To adhere to the social distancing measures, healthcare institutions suspended cancer screening and outreach programs and revised patient appointment schedules to maintain physical distance by limiting the number of patients that can move into the facility [6]. Other measures that indirectly affect cancer care delivery include task-shifting of health workers who previously delivered cancer care to be frontline health workers in health care facilities designated to handle COVID-19. These affect the delivery and access to cancer care services and exacerbate the barriers in access to care. These barriers include clinically significant delays, such as delay to seeking appropriate care, delay to reach an appropriate facility, and delay to receive adequate care at the facility.

Cancer care services are already underutilized even before the COVID-19 pandemic as seen in the literature and this has been attributed to various barriers. The underutilization and delay to seek for cancer care services have been attributed to barriers to access of cancer care services (screening, diagnosis, and treatment) such as a limited number health facilities offering cancer care services, long distances to healthcare facilities, transport costs, treatment costs and a limited number of cancer care providers. These barriers affect the utilization of cancer care services with the degree of utilization varying by cancer type. For example, a study done in two districts in Eastern Uganda in 2016 found that only 4.8% of women had ever screened for cervical cancer [8]. Screening allows for early detection of cancer and leads to better treatment outcomes and survival. At diagnosis, delays in attending the first oncology appointment, first treatment, and subsequent treatment schedules have been also reported. For example, a study conducted in Uganda in 2019 found that it takes a median of 84 days from the first presentation to consultation with a specialist, and a median of 34 days from specialist consultation to treatment initiation [9]. Delays in seeking care, attending first oncologist appointment, and in initiating cancer treatment have been found to have a negative impact on the treatment outcomes of cancer patients. These include a late-stage presentation at first diagnosis and progression of cancer to advanced stages. These cases of late presentation and advanced disease have been reported previously. In Kyadondo County, Uganda, about 89% of breast cancer patients present with stage III/IV disease, about 80% of cervical cancer patients present with stage III/IV disease [10].

Cancer care service delivery has been disrupted during the pandemic and effects are likely to vary per country. In Uganda, the suspension of the screening services and outreach programs has disrupted the efforts from sensitization campaigns on screening to treatment services, and it poses a big threat to the early detection of cancer cases. Therefore, we anticipate an increase in the proportion of patients who present with advanced stages of cancer in the period after lifting the COVID-19 pandemic lockdown measures that restrict access to cancer care services. The difference in days between oncologist appointments and treatment schedules requires that patients move between their homes and the cancer care centers. Considering the current lockdown measures, like limitations in public transport, the barrier to transportation to cancer care facilities has been exacerbated. The cost of public transport has almost doubled for most of the routes plied by public transport companies, in a bid to cover the cost of the reduced carrying capacity of their vehicles. This increases the financial burden of seeking care for cancer patients. The restrictions on economic activities have rendered the proportion of the people impoverished, and they are unable to meet the costs involved in seeking care. These dynamics on utilization and access to care can lead to an observation of a decrease in the numbers of detected cases of cancer during the COVID-19 pandemic period and an increase in cases detected during the period when lockdown measures affectting access to cancer services are fully lifted. Also, there can be variation in the proportion of individuals who present with advanced-stage cancer during pre, during, and post-COVID-19 pandemic era. Furthermore, there can be an increase in loss to follow up of patients after a diagnosis of cancer, poor adherence to treatment schedules, delays in access to cancer care after a diagnosis (first oncology appointment, initiation of treatment modalities), and increased mortality and reduced survival rates of patients. To understand how the COVID-19 pandemic measures in Uganda have impacted on the utilization and access to cancer care services, data collected by cancer registries can be used to quantify the effect.

Population-based cancer registries like the Kampala Cancer Registry are vital public health tools that can inform strategies for cancer control in a population and can be utilized to measure the impact of the COVID-19 pandemic on cancer care delivery and access in a population. Even before the COVID-19 pandemic, cancer registry data been providing information on cancer incidence, stage at diagnosis, and cancer survival and mortality for a given population that they serve.

## Conclusion

COVID-19 control measures are going to affect the delivery of and reduce access to cancer care services in Uganda. These measures can exacerbate the effects of previously reported barriers to delivery of and access to cancer care services such as the limited number of health facilities offering cancer care services, long distances to healthcare facilities, transport costs, treatment costs, and a limited number of cancer care service providers. These can widen the gap to access cancer care services in Uganda. Also, there can be a reduction in the number of new cancer cases registered in the population-based cancer registries. There is a need to synthesize data collected by cancer registries to measure the impact of COVID-19 control measures on delivery of and access to cancer care services in Uganda to inform the current and future response to epidemics while putting into context other diseases like cancer that have a high burden. There is a need to explore innovative strategies that can ensure continued delivery of cancer care services while observing COVID-19 pandemic control measures to avoid watering down the achievements that had been made in cancer control and other health concerns in Uganda.
